# Patient satisfaction and their determinants in outpatient department of a tertiary public hospital in Nepal: a cross-sectional study

**DOI:** 10.1186/s41687-024-00696-x

**Published:** 2024-02-28

**Authors:** Laxman Datt Bhatt, Sandhaya Ghimire, Kabita Khanal

**Affiliations:** 1 Faculty of Management Studies, Department of Healthcare Management, National Open College, Pokhara University, Lalitpur, Nepal; 2 Division of Population Health and Applied Health Sciences, Faculty of Medicine, Memorial University of Newfoundland, St. John’s, NL A1C 5S7 Canada; 3 Department of Pharmacy, Government of Nepal, Mental Hospital, Lalitpur, Nepal

**Keywords:** Patient satisfaction, Psychiatric hospital, Tertiary care hospital, Nepal

## Abstract

**Background:**

Patient satisfaction is a vital metric for assessing healthcare quality and delivering patient-centered care. It can predict service utilization patterns by determining healthcare users’ contentment with their providers. Consequently, evaluating patient satisfaction and its underlying factors is crucial to maintaining the quality of healthcare services. The present study aimed to assess patient satisfaction and its determinants in a tertiary care public hospital in Nepal. In this research, a cross-sectional design was employed to examine patient satisfaction within the Outpatient Department of Mental Hospital Lagankhel, Nepal. The study adopted a systematic random sampling approach for respondent selection, and stringent measures were implemented to uphold the validity and reliability of the collected data. To assess patient satisfaction comprehensively, the Patient Satisfaction Questionnaire-III (PSQ-III), developed by the RAND Corporation, was employed in conjunction with relevant sociodemographic variables. Utilizing mean scores and percentages, we calculated satisfaction levels across various dimensions. Additionally, a multinomial logistic regression analysis was conducted to investigate the relationships between patient satisfaction dimensions and sociodemographic characteristics.

**Results:**

This study encompassed perspective of 206 participants, with 57.3% representing patient relatives and 51% being male, median age of 32 years (standard deviation: 12.53). Notably, patients reported higher levels of satisfaction, particularly within the interpersonal relationship dimension, while the technical quality domain received comparatively lower satisfaction ratings. Multinomial logistic regression analysis underscored the significance of sociodemographic factors in shaping patient satisfaction, with age (*p* = 0.008), type of residence (*p* = 0.001), occupation (*p* = 0.0019), income status (*p* = 0.014), time to reach the healthcare facility (*p* = 0.013), and insurance enrollment status (*p* = 0.017) all demonstrating significant associations. These findings illuminate the intricate qualities of patient satisfaction within our healthcare context, offering actionable insights for enhancement and guiding the trajectory of future research endeavors.

**Conclusions:**

Overall patient expressed satisfaction with service provided by tertiary care hospital, however continuous improvement remains essential. Conducting large-scale, nationwide studies across hospital tiers is vital. This data-driven approach empowers policymakers to allocate resources effectively, inform decision-making, and enact policies that exceed patient expectations, fostering a healthcare system of unparalleled excellence.

## Background

Patient satisfaction serves as a compass that directs our understanding of healthcare quality, offering insight into the extent to which healthcare providers align with the expectations and wishes of their patients. It stands as a universal emblem of patient-centered care, shining its light across the global healthcare landscape [1]. Beyond a mere indicator, it functions as a vital metric, illuminating the path toward enhanced healthcare effectiveness and continual improvement [2]. While developed nations have long recognized it as a cornerstone of healthcare quality, in places like Nepal, where the daily struggle for essential necessities often takes precedence, the profound significance of patient satisfaction has yet to be fully charted [3].

The Outpatient Department (OPD) constitutes a pivotal nexus within the hospital infrastructure, representing the primary interface between patients and healthcare personnel. Furthermore, the OPD serves as a fundamental measurement reflecting the overall operational efficacy of the hospital, given its substantial utilization by the community [4]. In tandem with the evolving landscape of the healthcare sector, patient perspectives on healthcare have acquired widespread recognition as a fundamental benchmark for appraising healthcare quality [3, 4].

The global importance of studying patient satisfaction cannot be overstated, as it significantly influences the continuous improvement of healthcare systems worldwide. Despite Nepal’s dedication to providing comprehensive healthcare services with an emphasis on satisfying patients, achieving substantial progress remains a challenge. Furthermore, empirical research on patient satisfaction is notably limited within the context of Nepal’s healthcare landscape, particularly within tertiary care public hospitals [1]. This study aims to bridge this research gap by conducting a thorough evaluation of patient satisfaction levels among those receiving services at the Outpatient Department of Nepal’s government-run tertiary care Mental Hospital. The findings of this research hold the potential not only to illuminate the patient experience within tertiary care public hospitals but also to contribute to the broader global discourse on enhancing healthcare service quality [5].

## Methods

### Study design and sample

A hospital-based cross-sectional analytical study was executed from April 30 to May 24, 2022. We determined the sample size using the formula: *n* = *Z*^2^_(1 α/2)_ *pq*/*d*^2^ (where *Z*_(1−α/2)_ = 1.96 at 95% confidence; *p* = prevalence of patient satisfaction, *q* = 1−*p; d* = absolute allowable error. After literature review, we assumed 86% of patient satisfaction—as observed in a previous study from a similar setting [6]. Thus, *p* = 0.86; *q* = 0.14; *d* = 5%. The initial sample size calculated was 185, adding a 10% for non-response rate, resulting in a final sample size of 206 outpatients.

These participants were recruited from the Outpatient Department of Mental Hospital in Lalitpur, Nepal, the nation’s sole tertiary care psychiatric government hospital, consisting of 50 psychiatric beds and providing psychiatric services to over 200 daily OPD patients.

#### Sampling procedures and criteria for selection

Our research employed a systematic random sampling approach to select participants from the outpatient department (OPD). We employed a systematic random sampling approach to select patients for interviews from the OPD. Given a required sample size of 206 and a 21-day recruitment period, we calculated the kth number for patient selection each day using the formula kth number = (N/n) * k, where n represents the sample size (206), N denotes the total number of patients visiting the OPD in 21 days, and k signifies the day number. This allowed us to consistently select patients for interviews across the 21-day study period. If kth patient refused to participate, we extended the invitation to the next patient, continuing this process until we secured the targeted respondent for each day.

Inclusion criteria mandated that participants be first-time visitors to the OPD. To avoid response bias, our study deliberately excluded individuals who were healthcare facility staff or immediate family members seeking treatment, as well as patients attending for follow-up visits. Furthermore, patients presenting severe illness, impairment due to drugs or alcohol, or significant disabilities rendering them unable to participate effectively were also excluded. Identification of these medical conditions was facilitated by prior notification from the attending Psychiatrist in the OPD, streamlining the exclusion process during the interview phase and ensuring data accuracy.

#### Data collection

The data was collected using “CommCare” mobile-based digital data collection software and consent ensured on a separate paper form. A quantitative structured interview was used to record information. The questionnaire included the following parameters: (i) registration process, (ii) facilities in the waiting area such as clean toilets, drinking water, adequate space, (iii) services provided by the doctor, (iv) experience at the pharmacy, (v) behavior of staff with patients, (vi) availability of the prescribed medicines, (vii) demographic information of participants and (viii) out of pocket expenditure at healthcare facility for using services.

#### Data analysis

To assess overall satisfaction within specific domains, the summation of numerators for relevant variables was divided by the cumulative sum of their denominators, resulting in a satisfaction percentage. Subsequently, satisfaction levels were categorized as either “satisfied” (≤50%) or “unsatisfied” (≥49%). For Likert-type questions (as outlined in Table 3), a grading scale from 1 to 5 was employed, where responses of “strongly disagree” and “disagree” were consolidated into “no” satisfaction, “uncertain” responses remained unaltered, and “agree” and “strongly agree” were amalgamated into “yes” satisfaction. To determine the overall average rating for each respondent, individual ratings for each parameter were aggregated.

Data collected underwent download in MS-Excel format and subsequent exportation to Statistical Package for the Social Sciences Version 27.0 for comprehensive analysis utilizing both descriptive and inferential statistical methods. Descriptive statistics were presented as frequency and percentage distributions. The evaluation of associations between dependent and independent variables was conducted using chi-square tests and multinomial logistic regression. The survey instrument was meticulously constructed, incorporating validated patient satisfaction assessment tools (PSQ-III and PSQ-18, developed by the RAND Corporation). Contextual sociodemographic characteristics were thoughtfully integrated into the survey instrument. Statistical significance was defined as a *p*-value below 0.05, signifying a significant association.

## Results

Table 1 presents the sociodemographic characteristics of the participants, consisting of 206 individuals. The majority of respondents were patients’ friends and relatives, comprising 57.3% (n = 118) of the sample, while the remaining respondents were patients themselves. More than half of the participants were male (51%, n = 105). The median age of the respondents was 32 years (SD = 12.53), with an interquartile range of 17.25. Respondents were categorized into two age groups based on the median age, wherein the age group of 32 and below constituted more than half of the population, while the age group of 32 and above accounted for approximately half of the participants (51%, n = 105 and 49%, n = 101, respectively).

**Table 1:** Sociodemographic characteristics of participants (n = 206)

Characteristics	Frequency (n = 206)	Percentage (%)
**Interviewee**		
Patient	88	42.7
Patients’ friend	118	57.3
**Gender**		
Male	105	51
Female	101	49
**Age**		
≤32	105	51
>32	101	49
*Median age* *=* *32, SD* *=* *12.53, Inter Quartile Range (IQR)* *=* *17.25, Maximum* *=* *74, Minimum* *=* *14*		
**Ethnicity**		
Brahmin/Chhetri	90	43.7
Janajati	90	43.7
Dalit	18	8.7
Madhesi	3	1.5
Muslim	1	0.5
Others	4	1.9
**Religion**		
Hindu	152	73.8
Buddhist	33	16.0
Christian	15	7.3
Muslim	2	1.0
Others	4	1.9
**Residence**		
Urban	109	52.9
Rural	97	47.1
**Education**		
Literate	29	14.1
Illiterate	20	9.7
Primary level	34	16.5
Secondary level	65	31.6
Higher education	58	28.2
**Marital status**		
Married	140	68.0
Unmarried/Single	62	30.1
Widowed	4	1.9
**Occupation**		
Agriculture	44	21.4
Business	46	22.3
Homemaker	46	22.3
Service	31	15.0
Others	39	18.9
**Wealth quintile (n** **=** **153)**		
Lowest	41	26.79
Second	55	35.94
Middle	35	22.87
Fourth	8	5.22
Highest	14	9.15
*Median income* *=* *19,000, Inter Quartile Range (IQR* *=* *18,000, Minimum* *=* *1000, Maximum* *=* *60,000*		
**Time to reach health facility**		
Less than 30 min	26	12.62
30–60 min	85	41.26
More than 1 h	95	46.12
**Health insurance**		
No	163	79.1
Yes	43	20.9

Approximately 44% (n = 90) of the participants identified as Brahmin/Chhetri, which was equivalent to the proportion of Janajati ethnic group respondents. Less than 1% (0.5%, n = 1) of the participants identified as Muslim.

Among the participants, a majority of participants were from an urban area (52.9%, n = 109) and more than one-fifth of them had completed secondary level education (31.6%, n = 65), while 9.7% (n = 20) were illiterate. The majority of respondents (68.0%, n = 140) were married, and an approximately equal number of respondents were engaged in business, household work, and agriculture (22.3%, n = 46; 22.3%, n = 46; and 21.4%, n = 44, respectively). Among the participants, nearly three quarters (73.8%, n = 152) reported following Hinduism, and only 1% (n = 2) identified as Muslim.

Of the total respondents, 53 participants did not provide their actual monthly household income or were hesitant to answer the question. Among the remaining participants, 35.94% (n = 74) fell into the second quartile. Almost half of the respondents (46.12%, n = 95) took more than an hour to reach the health facility. A significant majority (79.1%, n = 163) did not enroll in the national health insurance program, while 20.9% (n = 43) of the respondents had enrolled in health insurance and received benefit packages from the health facility.

Table 2 shows the information regarding source of information to visit health facility, reason of visit, waiting time, and overall satisfaction of respondents with OPD time. Most of the respondents reported their immediate friends, relatives, and family members as their source of information about the hospital (82.5%, n = 170), and majority of the responded reported quality service as the major reason of visiting the health facility (42.7%, n = 88). More than half (54.9%, n = 11) of the respondents visited other health facility prior visiting this facility and among the visited more than half (54.86, n = 62) went to private health facility.

**Table 2:** Hospital related information (n = 206)

Characteristics	Frequency (n = 206)	Percentage (%)
**Source of information to visit hospital**		
Friends/Family	170	82.5
Internet	11	5.3
Newspaper	12	5.8
Others	13	6.3
**Main reason to visit**		
Good quality	88	42.7
Near to home	20	9.7
Relative suggestion	70	34.0
Referred	14	6.8
Others	14	6.8
**Other hospital before**		
Yes	113	54.9
No	93	45.1
**Place of visit (n** **=** **113)**		
Private	62	54.86
Public	51	45.14
**Visited due to proximity**		
Yes	49	23.8
No	157	76.2
**Waiting time**		
≤60 min	139	67.5
>60 min	67	32.5
*Median waiting time* *=* *61.88, IQR* *=* *100, Maximum time* *=* *4-h, Minimum time* *=* *1 min*		
**Satisfied with OPD time**		
Yes	179	86.9
No	27	13.1

Three fourths of the respondents (76.2%, n = 157) said proximity was not the reason of visiting this facility. Median waiting time for all medical procedures was 61.88 min with an interquartile range of 100 (Max = 1 h; Min = 1 min) while many of the respondents (67.5%, n = 139) had waiting time below and average to 60 min. Most of the visiting patients (86.9%, n = 179) responded with their satisfaction on OPD time while 13.1% (n = 27) responded said dissatisfaction on OPD timing.

Table 3 summarizes the satisfaction level of the participants for each item of PSQ-18. In the domain of accessibility and availability, about 75% (n = 154, X̄ = 4.03; SD: 1.10) reported that the registration procedure was fully perfect while respondents reported waiting time as a major dissatisfaction among the above mentioned dimension (25.2%; n = 52; X̄ = 3.49; SD = 1.28). In physical environment 81.6% respondents (X̄ = 4.21; SD = 0.93) reported that hospital had clean area and checkup room are good. In the interpersonal relationship domain, the satisfaction scale ranges from 4.08 to 4.18, the majority of the respondents were fully satisfied with doctors’ behavior during the medical checkup followed by hospitals, and other administrative and technical staffs behavior however the time allocated by doctors to them for treatment procedure was not satisfactory (13.6%; n = 28; X̄ = 4.08; SD = 1.17). More than half (66.0%; n = 136) respondents were satisfied with doctors communication for decision making process and satisfaction score ranges from 3.87 with standard deviation of 1.13 in this dimension.

**Table 3:** Satisfaction of patient by each item of the patient satisfaction questionnaire (n = 206)

Item	Question	No (strongly disagree + disagree) %	Uncertain %	Yes (strongly agree + agree) %	Mean score	S.D
**Accessibility and convenience**						
1.	Location of the hospital	41 (19.99)	28 (13.6)	137 (66.5)	3.78	1.21
2.	Registration procedure	23 (11.2)	29 (14.1)	154 (74.8)	4.03	1.10
3.	Waiting time	52 (25.2)	36 (17.5)	118 (57.3)	3.49	1.28
4.	Waiting area	40 (19.4)	33 (16.0)	133 (64.6)	3.73	1.21
5.	Information accessibility	28 (13.6)	25 (12.1)	153 (74.3)	3.97	1.20
**Physical environment**						
6.	Cleanliness of hospital area	37 (18.0)	32 (15.5)	137 (66.5)	3.72	1.15
7.	Level of noise in hospital	35 (17.0)	38 (18.4)	133 (64.6)	3.69	1.11
8.	Cleanliness of checkup room	11 (5.3)	27 (13.1)	168 (81.6)	4.21	0.93
**Interpersonal relationship**						
9.	Behavior of hospital staff	19 (9.2)	22 (10.7)	165 (80.1)	4.18	1.05
10.	Doctors’ behavior	18 (8.7)	12 (5.8)	176 (85.4)	4.36	0.99
11.	Doctors time	28 (13.6)	25 (12.1)	153 (74.3)	4.08	1.17
**Communication and decision making**						
12.	Communication with doctor	29 (14.1)	41 (19.9)	136 (66.0)	3.87	1.13
**Financial aspect**						
13.	Cost paid for medicine	40 (19.4)	43 (20.9)	123 (59.7)	3.59	1.19
14.	Cost paid to health institution	28 (13.6)	40 (19.4)	138 (67.0)	3.77	1.12
15.	Fee paid vs quality of care	23 (11.2)	32 (15.5)	151 (73.3)	4.02	1.075
**Technical quality**						
16.	Availability of drugs	33 (16.0)	36 (17.5)	137 (66.5)	3.83	1.20
17.	HR quality and hospital	27 (13.1)	39 (18.9)	140 (68.0)	3.81	1.083
18.	Treatment procedure	21 (10.2)	21 (10.2)	164 (79.6)	4.14	1.04

Majority of participants (73.3%; n = 151; X̄ = 4.02; SD = 1.075) said the total cost paid to the hospital for treatment and medication process was satisfactory while the cost paid for medicine purchase was expensive. The financial aspect satisfaction score ranges from 3.59 lowest to 4.02 as the highest score of satisfaction. Mean satisfaction score in technical quality ranges from (3.83–4.14) with the highest score in treatment procedure (79.6%; n = 164; X̄ = 4.14; SD = 1.04) followed by availability of drugs (3.83) and the lowest in Human resource quality at hospital.

### Inferential statistics

Table 4 presents the association between sociodemographic factors and patient satisfaction Out of 11 characteristics, five variables were found statistically significant (*p* < 0.05). Age group of the respondents was statistically significant with patient satisfaction (*p* = 0.0008). Similarly respondent from rural area were 5 times more likely to be satisfied with compared to urban area (*p* = 0.001; OR = 4.871; 95% CI = 2.150–11.038). Occupation of the respondents was also observed as a significant factor for patient satisfaction. Respondents with agriculture as a major occupation were nearly 0.448 times more likely to be satisfied than other occupations (*p* = 0.019; OR = 0.448; 95% CI = 0.147–1.364. With time to reach a health facility, respondents who reached HF less than 30 min were 3.890 times more likely to be satisfied with hospitals service related satisfaction (*p* = 0.013; 95% CI = 1.282–11.799) and those enrolled in national health insurance program were 0.214 times more likely to be satisfied with compared to non-enrolled (*p* = 0.017; OR 0.214; 95% CI = 0.057–0.086).

**Table 4:** Association between sociodemographic factors and patient satisfaction (n = 206)

Characteristics	Unsatisfied	Satisfied	*P*-value	OR	95% CI
**Age**					
≤32	59 (56.2)	46 (43.8)	0.008**	3.073	1.302–7.253
>32	38 (37.6)	63 (62.4)			
**Gender**					
Male	50 (47.6)	55 (52.4)	0.876		
Female	47 (46.5)	54 (53.5)			
**Ethnicity**					
Brahmin/Chhetri	44 (48.9)	46 (51.1)	0.588		
Dalit	6 (33.3)	12 (66.7)			
Janajati	44 (48.9)	46 (51.1)			
Madhesi	2 (66.33)	1 (33.33)			
Muslim	0 (0.0)	1 (100)			
Others	1 (50.0)	1 (50.0)			
**Religion**					
Hindu	68 (44.7)	84 (55.3)	0.338		
Buddhist	15 (45.5)	18 (54.5)			
Christian	11 (73.3)	4 (26.7)			
Muslim	1 (50.0)	1 (50.0)			
Others	2 (50.0)	2 (50.0)			
**Residence**					
Rural	53 (54.6)	44 (45.4)	0.001***	4.871	2.150–11.038
Urban	44 (40.4)	65 (59.6)			
**Education**					
Higher education	29 (50.0)	29 (50.0)	0.550		
Illiterate	12 (60.0)	8 (40.0)			
Literate	11 (37.9)	18 (62.1)			
Primary level	17 (50.0)	17 (50.0)			
Secondary level	28 (43.1)	37 (56.9)			
**Marital status**					
Married	63 (45.0)	77 (55.0)	0.092		
Unmarried	33 (53.2)	29 (46.8)			
Widow	1 (25.0)	3 (75.0)			
**Occupation**					
Agriculture	20 (45.5)	24 (54.5)	0.019*	0.448	0.147–1.364
Business	16 (34.8)	30 (65.2)			
Housewife	17 (37.0)	29 (63.0)			
Others	26 (66.7)	13 (33.3)			
Service	18 (58.1)	13 (41.9)			
**Income**					
Lowest	19 (46.34)	22 (53.65)	0.014*	1.152	0.339–3.915
Second	23 (41.81)	32 (58.18)			
Middle	18 (51.43)	17 (48.57)			
Fourth	4 (50.0)	4 (50.0)			
Highest	6 (42.85)	8 (57.14)			
**Time to reach HF**					
Less than 30 min	14 (53.8)	12 (46.2)	0.013*	3.890	1.282–11.799
30–60 min	44 (51.8)	41 (48.2)			
More than 1 h	39 (41.1)	56 (58.9)			
**Health insurance**					
Yes	19 (47.9)	24 (52.1)	0.017*	0.214	0.057–0.086
No	78 (44.2)	85 (55.8)			

Figure in parenthesis shows percentage

*p-value less than 0.05 at 5% level of significance

**p-value less than 0.01 at 5% level of significance

***p-value less than or equal to 0.001 at 5% level of significance

Table 5 summarizes the association of hospital-related factors with patient satisfaction. Out of eight variables, only 3 variables showed significant association with patient satisfaction. Quality of service was significantly associated with patient satisfaction and other factors including referral to other facilities, relative suggestion, and nearby homes as a reason of visit were 1.570 times more likely to be dissatisfaction factors compared with quality of care (*p* = 0.020; OR = 1.570; 95% CI = 0.825–2.989). Visit to other health facility prior to visit this facility was also significantly associated with patient satisfaction (*p* = 0.043). It was found that those visited other health facility before were 1.7 times more likely to be satisfied with hospitals services (OR = 1.769; 95% CI = 1.016–3.080) and existing OPD time was also found to be significantly associated with patient satisfaction factor. It was found that those who are fine with current OPD service are 8 times more likely to be satisfied with hospital services compared to one, with those who did not. (*p* = 0.001; OR = 8.159; 95% CI = 2.709–24.576).

**Table 5:** Association between hospital related factors and patient satisfaction (n = 206)

Characteristics	Unsatisfied	Satisfied	*P*-value	OR	95% CI
**First visit**					
Yes	29 (56.9)	22 (43.1)	0.107		
No	68 (43.9)	87 (56.1)			
**Knowledge of hospital**					
Friends/family	75 (44.1)	95 (55.9)	0.220		
Newspaper/radio	6 (50.0)	6 (50.0)			
Internet	7 (63.6)	4 (36.4)			
Others (Self)	9 (69.2)	4 (30.8)			
**Main reason to visit**					
Good quality	41 (46.6)	47 (53.4)	0.020*	1.570	0.825–2.989
Near	11 (55.0)	9 (45.0)			
Referred	9 (64.3)	5 (35.7)			
Relatives’ suggestion	25 (35.7)	45 (64.3)			
Others	11 (78.6)	3 (21.4)			
**Other HF before**					
Yes	46 (40.7)	67 (59.3)	0.043*	1.769	1.016–3.080
No	51 (54.8)	42 (45.2)			
**HF visited before (n** **=** **113)**					
Public	19 (37.3)	32 (62.7)	0.104		
Private	27 (43.5)	35 (56.5)			
**Visit due to proximity**					
Yes	22 (44.9)	27 (55.1)	0.725		
No	75 (47.8)	82 (52.2)			
**Waiting time**					
1 h and less	66 (47.5)	73 (52.5)	0.870		
More than 1 h	31 (46.3)	36 (53.7)			
**OPD time**					
Yes	74 (41.3)	105 (58.7)	0.001**	8.159	2.709–24.576
No	23 (85.2)	4 (14.8)			

Figure in parenthesis shows percentage

*p-value less than 0.05 at 5% level of significance

**p-value less than 0.01 at 5% level of significance

***p-value less than or equal to 0.001 at 5% level of significance

Table 6 presents the information of the respondent regarding re-utilization of hospital services. It was found that almost all (93.2%; n = 192) respondents would be returning to this hospital for further follow-up and treatment procedures and 5.8% (n = 12) respondents were not sure about the further revisit plan and 1% would not return back to this facility for further health care service reutilization. Similarly, the responded reported that almost all (92.7%; n = 191) would recommend Mental hospital Lagankhel to others those requiring psychiatric healthcare services, 1% (n = 2) would not recommend to others later on and 6.3% (n = 13) respondents were not sure about recommending to others.

**Table 6:** Reutilization of service (n = 206)

Variables	Frequency (n = 206)	Percentage (%)
**Willingness to return**		
Yes	192	93.2
No	2	1.0
Not sure	12	5.8
**Willingness to recommend others**		
Yes	191	92.7
No	2	1.0
Not sure	13	6.3

Table 7 summarizes respondents’ qualitative responses on good aspects, bad aspect, and recommendations for hospital by patients to improve existing healthcare services. Regarding good aspects of the hospital, more than half (67.89%, n = 129) responded hospital (provider) has a good aspect which includes quality of care provided by doctors, doctors, counselling, and pharmacy service with medicines availability and 26.31% (n = 53) and 5.78% (n = 12) responded good aspects were more related with facility and logistic, respectively.

**Table 7:** Qualitative data from respondents’ aspects (n = 206)

Variables	Provider	Facility	Logistic
Good aspect (n = 190)	129 (67.89)	50 (26.31)	11 (5.78)
Bad aspect (n = 152)	30 (19.73)	52 (34.21)	70 (46.06)
Suggestions (n = 128)	36 (28.13)	74 (57.82)	18 (14.06)

Similarly, nearly half the respondent (46.06%; n = 70) said the bad aspects of hospitals were more related with logistics including waiting area, sanitation, poor hygiene, and unmanaged que system. Regarding respondents suggestions to health facilities, more than half of the respondents (57.82%, n = 74) reported that the hospital could improve their existing facilities including increment in IPD beds, token system for line management, availability of all medicines prescribed by doctors, doctors time to patients and availability of all services in the hospital with less number of referral to external health facility, while 28.13% (n = 36) reported suggestions related to provides including doctors, paramedics their behaviors, counselling skills and remaining respondent reported logistic management as a suggestion for health facility to improve in the upcoming days.

## Discussion

The present study aimed to assess patient satisfaction with various components of healthcare in government health facilities providing tertiary care in Lalitpur district, Nepal. Limited studies have investigated patient satisfaction in this context, and thus there is a dearth of data for comparison with existing evidence. However, the findings of this study could be used to enhance the quality of healthcare if transformed into actionable interventions. According to a similar patient satisfaction study conducted at Nepal Medical College Teaching Hospital, the satisfaction level with outpatient department (OPD) services was 52.9%, which is lower than the overall satisfaction rate (74.8%) observed in that study [1]. Technical quality, physical environment, and some components of accessibility and convenience had lower satisfaction scores [7–9], potentially due to factors such as disease status, unavailability of nearby psychiatric care centers, high patient flow, and limited doctors and paramedics [10, 11].

In terms of patient satisfaction domains assessed by the Likert scale, the interpersonal relationship domain had the highest satisfaction score (85.4%), followed by physical environment and accessibility and convenience (81.6% and 74.8%, respectively). However, a study conducted at Western Regional Hospital in Pokhara, Nepal, showed that the highest satisfaction level was observed in the accessibility and convenience domain [12]. This disparity in findings within a similar healthcare setting could be attributed to differences in sample size, data collection tool, and specialized care provided by both tertiary care hospitals [9, 13]. A study conducted in a private hospital in India demonstrated that approximately 91% of patients were satisfied with the interpersonal relationship domain, specifically with regard to doctor time allocation to patients [14], while in our study, the proportion of satisfied patients in this dimension was about 75%. This difference may be due to doctors spending less time with patients at our study site and not making adequate efforts to establish a strong doctor-patient relationship in public hospitals.

Regarding the financial aspect of patient satisfaction factors, a study on eye services at Nepal Medical College showed that 76.8% of patients were satisfied with the fees paid and quality of care provided by the hospital [15]. In our study, 73.3% of patients expressed overall satisfaction with the fees paid and quality of care provided by the hospital, which is consistent with the findings of the previous study. This alignment could be due to the availability of government-owned psychiatric care hospitals and the provision of affordable healthcare services and medicines to patients enrolled in the social health insurance program.

Our study revealed that age, type of residence, occupation, time to reach the hospital from home, and insurance enrollment status were the main predictors of patient satisfaction. A systematic review of patient satisfaction determinants worldwide indicated that age and distance to healthcare facilities were the most significant and consistent predictors of patient satisfaction [16–19]. This may be due to differences in service and treatment perceptions between older and younger patients, with older patients being more comfortable with existing healthcare services. Our study did not identify a significant association between gender and satisfaction, which is supported by various previous studies [16, 20], although other research has reported conflicting findings [1, 7, 10, 17]. Additionally, our study found that education level, ethnicity, religion, and marital status were not significantly associated with patient satisfaction, while other studies have reported significant associations between these variables and patient satisfaction [3, 16, 20]. These differences may be due to the diversity of ethnic group doctors in hospitals, patients’ perspectives on the services provided by healthcare facilities, communication and decision-making skills of clinicians, and the technical quality of the facility, providers, and logistics items, without considering religious factors in healthcare services.

### Strengths and limitations

One of the standout strengths of this study lies in its comprehensive evaluation of patients within the outpatient departments (OPD) of tertiary care hospitals in Nepal. This approach is of paramount importance as it affords an intricate and in-depth analysis of the healthcare services’ quality within these crucial institutions. Moreover, the utilization of a validated questionnaire further bolsters the study’s robustness, guaranteeing the reliability and validity of the amassed data.

However, the study does have its limitations. The fact that patient interviews were conducted within the healthcare facility introduces the potential for social desirability bias, which remains beyond the study’s control. Additionally, while the study identifies patient dissatisfaction with certain dimensions of healthcare service quality, the quantitative nature of the research may not fully capture the nuanced aspects of this dissatisfaction. To address this limitation, a qualitative study could be envisaged to glean more profound insights.

Furthermore, it is essential to acknowledge that patient responses may be influenced by individual personality traits and varying perceptions, which may, in turn, impact the generalizability of the findings. Lastly, it’s crucial to recognize that this study’s scope is confined to tertiary care public healthcare facilities. A comparative study encompassing both public and private hospitals could furnish a more comprehensive analysis of healthcare service quality, offering a broader perspective on the matter.

## Conclusions

In summary, our findings underscore the resilience of government tertiary care hospitals in providing commendable healthcare services despite inherent constraints in terms of human resources, infrastructure, and logistics. While the healthcare facilities have undoubtedly made significant strides, there is still ample room for improvement to achieve the pinnacle of service quality. Our study revealed that patients expressed the least satisfaction with the technical quality dimensions of healthcare, highlighting an area ripe for enhancement. Conversely, patients reported the highest levels of satisfaction within the interpersonal domain, affirming the importance of compassionate care.

Crucially, sociodemographic factors, including age, residential area, occupation, travel time to healthcare facilities, and enrollment in social health insurance, emerged as significant influencers of patient satisfaction. These factors should be considered in tailoring healthcare services to diverse patient needs. It is noteworthy that while interpersonal relationships received the highest mean satisfaction scores, accessibility and convenience lagged behind. Therefore, targeted interventions focusing on improving registration processes, waiting times, queuing systems, sanitation, waiting area management, and enhancing information accessibility for patients are imperative.

Looking ahead, we advocate for ongoing large-scale studies and internal quality enhancement initiatives to be conducted regularly across hospitals at various levels throughout the country. These endeavors will provide a comprehensive and evolving understanding of patient satisfaction, contributing to the continuous improvement of healthcare services nationwide.

## Data Availability

The datasets used and analysed during the current study are available from the corresponding author on reasonable request.
